# Advances in Tinnitus and Hearing Disorders

**DOI:** 10.3390/brainsci15060553

**Published:** 2025-05-23

**Authors:** Alessandra Fioretti

**Affiliations:** European Hospital, 00149 Rome, Italy; abfioretti@gmail.com

Tinnitus is generally defined as the perception of sound in the absence of a corresponding external acoustic stimulus [1], but its definition in recent years has been extended to possible emotional and cognitive components [2]. For this reason, new definitions of “auditory hallucinosis” [3] and “tinnitus disorder” were introduced [4]. Messina et al. suggested that tinnitus is an auditory dysperception that can be classified as auditory hallucinosis connected to the phenomenon dysneuro-plasticity, “almost always” resulting from an organic cochlear peripheral lesion [3]. Persistent tinnitus can lead to significant psychological distress, accompanied by depression and anxiety, which seriously affects quality of life and causes difficulties in sleeping, concentrating, and emotional well-being, even resulting in a functional disability, as in the case of tinnitus disorder [4]. The prevalence of all types of tinnitus reported in the literature has a mean rate of 15% [5]. Severe tinnitus affects an average of 2.3% of cases and increases with age [6]. Many patients with tinnitus have some degree of hearing loss, while other patients with significant hearing loss do not have tinnitus. In recent years, the discovery of cochlear synaptopathy revealed that the role of hidden hearing loss in the presence of tinnitus is not associated with audiometric hearing loss. Objective diagnostic markers such as extended high-frequency audiometric thresholds, otoacustic emissions, speech in noise, and auditory brainstem response are now suggested in the evaluation of some patients with tinnitus and apparently normal hearing [7]. Common causes have been identified as the potential etiology of tinnitus, such as noise-induced hearing loss, presbycusis, Meniere’s disease, infections, head trauma, acoustic neuroma, otosclerosis, and temporo-mandibular disorders, but in many cases, the etiology of tinnitus is idiophatic [1]. Many treatment options for tinnitus are available including hearing aids, cochlear implants, sound generators, auditory stimulation approaches, tinnitus retraining therapy (TRT), cognitive–behavioral therapy (CBT), pharmacological treatment, neurofeedback, brain stimulation, bimodal stimulation, and Internet- and app-based digital approaches [8]. According to the Tinnitus Guidelines [9,10,11], pharmacological interventions such as antidepressants, anticonvulsants, and antianxiety medications are not recommended. Instead, cognitive–behavioral therapy (CBT) and the use of hearing aids are considered effective. The research on tinnitus and hearing disorders is now implementing new technologies to better understand their pathophysiological mechanisms, which are not well established, and to develop new clinical instruments for diagnosis and therapy. In recent decades, clinical and biochemical markers (including imaging techniques and genetics), as well as teleaudiology, have emerged as promising tools for hearing disorders and tinnitus [12,13,14]. This Editorial refers to the Special Issue “Advances in tinnitus and hearing disorders”, which highlights new opportunities and challenges for advancing in pathophysiological research on tinnitus, focusing on its technological advancements and management instruments. Eighteen manuscripts were submitted for consideration for the Special Issue, and all of them were subject to a rigorous review process. In total, eight papers were finally accepted for publication and included in this Special Issue (six articles, one perspective, and one review). The contributions are listed below:

Norena (contribution 1) delved into the analogy between tinnitus and pain from a phenomenological point of view to understand how tinnitus can, like chronic pain, generate an existential “crisis”, which may go as far as leading to the collapse of the subject. The research suggests that tinnitus may be compared with the phenomenon of pain based on the way it is experienced. Although the analogy between tinnitus and pain has often been made in the literature, it has been limited to a parallel concerning the putative physiopathological mechanisms.

Bourez et al. (contribution 2) aimed to propose a clinical simulation method of tinnitus to strengthen students’ skills and confidence in tinnitus evaluation and management. This simulation technique allows a student playing the role of an audiologist to practice standard psychoacoustic measurements of tinnitus in a similar mode to that of testing on a real tinnitus patient. The findings reveal the opportunity for audiology educators and other healthcare educators to train students in tinnitus evaluation and management, encouraging critical reflection and cultivating greater empathic care towards patients.

Jedrzejczak et al. (contribution 3) studied chatbot use in relation to tinnitus. Their study looked at the effects that AI technology advances have on tinnitus patients and audiological practice. Chatbot use is currently a very hot topic in medical research, and studies have begun to explore issues such as the efficacy of AI tools in audiology. Their work’s main goal was to outline the accuracy of ChatGPT’s (version 3.5) responses to a defined set of 10 questions about tinnitus and its responses to the same questions after another 3 and 6 months. ChatGPT’s responses were rated as satisfactory or better by the authors, and the authors did not find any potentially harmful errors. However, some omissions could be considered misleading, and some limitations were due to the lack of solid references.

Lauriello et al. (contribution 4) examined the role of executive functions (EFs) in preschool children aged between 2 and 6 years with sensorineural hearing loss (SNHL) and in patients with specific language impairment (SLI). The results demonstrated that the skills investigated were found to be deficient in both SNHL and SLI patients. Children with SNHL showed a clear deficit in flexibility and working memory, whereas children with SLI had greater problems in self-regulation and management of waiting for gratification. Additionally, the authors suggested to start targeted exercises based on specific deficient skills as part of the rehabilitation program.

Inguscio et al. (contribution 5) experimentally examined the associations between electroencephalographic (EEG) and psycho-audiological variables (Symptoms Checklist-90-Revised; State-trait Anxiety Inventory; Big Five Inventory-10; Tinnitus Handicap Inventory; Tinnitus Questionnaire 12-I; and Khalfa’s Hyperacusis questionnaire) in 12 tinnitus patients and 7 controls during an audio cognitive task and at rest. In the tinnitus patients, frontal beta activity positively correlated with hyperacusis, parietal activity, and trait anxiety. The positive correlation observed between beta activity in the frontal areas and hyperacusis could decline into an auditory-linked neurophysiological vigilance. The findings of the study indicate that an integrated approach to treatment, for example combining psychological therapy, like cognitive behavioral therapy, and neurofeedback, may be more beneficial for tinnitus patients.

Lee et al. (contribution 6) aimed to illustrate how computer-mediated counseling can contribute to the alleviation of the negative emotional impact of tinnitus. Fifteen participants with tinnitus were assigned to online group counseling (attending counseling via an online conferencing system for 6 sessions over 2 weeks) and twenty-one participants with tinnitus to video-based counseling (watching educational videos on tinnitus for 12 sessions over 2 weeks). The research shows significant improvements in average emotional aspect-related scores in both groups.

Bal et al. (contribution 7) investigated the effectiveness of the Eye Movement Desensitization and Reprocessing (EMDR) method on chronic subjective tinnitus. According to their findings, the difference between tinnitus severity levels pre- and post-EMDR in tinnitus patients was found to be statistically significant. After EMDR, a decrease was observed in the symptoms of the participants. EMDR can be used in the treatment of tinnitus and as an alternative treatment with significant effect on reducing and improving tinnitus. The study also discovered that EMDR is more efficacious in treating tinnitus than the masking method.

Perin et al. (contribution 8) analyzed the literature on neural bases of tinnitus and astrocytes implicated in both neural responses to inflammation and plasticity regulation. A systematic literature review was performed on the possible roles of astrocytes in the neural mechanisms leading to acute and chronic tinnitus, in particular in tinnitus comorbidities, inflammation, and the emerging role of astrocytes as regulators of neuronal context. The research shows that astrocytes are an understudied cell in the context of tinnitus, and recent results in other neuroscience fields suggest that their properties could explain some of the knowledge gaps about tinnitus.
